# Predicting clinical outcomes at hospital admission of patients with COVID-19 pneumonia using artificial intelligence: a secondary analysis of a randomized clinical trial

**DOI:** 10.3389/fmed.2025.1561980

**Published:** 2025-05-02

**Authors:** Caio César Souza Conceição, Camila Marinelli Martins, Mayck Medeiros Silva, Hugo Caire de Castro Faria Neto, Davide Chiumello, Patricia Rieken Macedo Rocco, Fernanda Ferreira Cruz, Pedro Leme Silva

**Affiliations:** ^1^Laboratory of Pulmonary Investigation, Institute of Biophysics Carlos Chagas Filho, Federal University of Rio de Janeiro, Rio de Janeiro, Brazil; ^2^AAC&T Research Consulting LTDA, Curitiba, Brazil; ^3^Laboratory of Immunopharmacology, Oswaldo Cruz Institute (Fiocruz), Rio de Janeiro, Brazil; ^4^Department of Health Sciences, University of Milan, Milan, Italy; ^5^Anaesthesia and Intensive Care, San Paolo University Hospital, Milan, Italy; ^6^Coordinated Research Center on Respiratory Failure, University of Milan, Milan, Italy

**Keywords:** COVID-19, biomarkers, machine learning, LASSO, CombiROC, clinical improvement

## Abstract

**Background:**

Predicting clinical improvement after hospital admission in patients with COVID-19 is crucial for effective resource allocation. Machine-learning tools can help identify patients likely to show clinical improvement based on real-world data. This study used two approaches—least absolute shrinkage and selection operator (LASSO) and CombiROC—to identify predictive variables at hospital admission for detecting clinical improvement after 7 days.

**Methods:**

A secondary analysis was conducted on the modified intention-to-treat placebo group from a previous randomized clinical trial (RCT, NCT04561219) of patients with COVID-19. The analysis assessed clinical, laboratory, and blood markers at admission to predict clinical improvement, defined as a two-point increase on the World Health Organization clinical progression scale after 7 days. LASSO and CombiROC were used to select optimal predictive variables. The Youden criteria identified the best threshold for different variable combinations, which were then compared based on the highest area under the curve (AUC) and accuracy. AUCs were compared using DeLong’s algorithm.

**Results:**

Overall, 203 patients were included in the analysis, and they were divided into two groups; clinical improvement (*n* = 154) and no clinical improvement (*n* = 49). The median age was 55 years (interquartile range, 46–66 years); 65% were male. LASSO identified three predictive variables (SaO_2_, hematocrit, and interleukin [IL]-13) with high sensitivity of 98% (95% confidence interval [CI], 92–100%) but low specificity of 26% (95% CI, 10–48%) for clinical improvement. CombiROC selected a broader set of variables (T cell–attracting chemokine, hemoglobin, hepatocyte growth factor, hematocrit, IL-3, PDGF-BB, RANTES, and SaO_2_), achieving balanced sensitivity of 82% (95% CI, 69–91%) and specificity of 74% (95% CI, 49–91%). LASSO and CombiROC showed comparable accuracy (82 and 80%, respectively) and similar area under the ROC curves (LASSO: AUC, 0.704; 95% CI, 0.571–0.837; CombiROC: AUC, 0.823; 95% CI, 0.708–0.937; *p* = 0.185).

**Conclusion:**

For patients hospitalized with COVID-19 pneumonia, predictive variables identified by LASSO and CombiROC analyses demonstrated similar accuracy and AUCs in predicting clinical improvement. LASSO, with fewer variables (SaO_2_, hematocrit, and IL-13) showed high sensitivity but low specificity, whereas CombiROC’s broader selection of variables provided balanced sensitivity and specificity for predicting clinical improvement.

**Clinical trial registration:**

Brazilian Registry of Clinical Trials (REBEC) number RBR-88bs9x and ClinicalTrials.gov number NCT04561219.

## 1 Introduction

The COVID-19 pandemic reshaped thinking around prevention and treatment strategies for emerging diseases, as well as approaches to health resource allocation (1). In most cases, the symptoms of COVID-19 are mild and improve within days, but a small subgroup of patients develop severe disease, marked by significant multi-organ dysfunction (2). Predicting whether a patient will progress to clinical improvement or deterioration can help with the allocation of equipment and human resources; however, advances in this field have been modest. Worldwide, clinicians and researchers have been developing prognostic tools, including risk scores, biomarker screening, and machine-learning models, to better predict the clinical course of COVID-19 and the outcomes (3, 4). Despite the promise of artificial intelligence (AI) in this domain, appropriate statistical comparisons between different predictive methods are still lacking (5).

Working with numerous predictive variables is challenging with traditional analyses when seeking robust predictions. In this context, least absolute shrinkage and selection operator (LASSO) regression has emerged as a tool for predicting outcomes in patients with COVID-19 (4, 6–10). A key advantage of LASSO is its capacity to reduce the number of explanatory variables in a model and to address multicollinearity within the data (11). Another approach, CombiROC, offers a flexible method for managing complex data in discriminative analyses (12). A key advantage of CombiROC is the combinatorial analysis and ROC curves. Both methods have been used in medicine and other areas for many decades, but they generally lack an easy-to-use interface that researchers without programming skills can use to analyze data and create plots. With CombiROC, it is possible to select the combinations of optimal markers and obtain immediate visual feedback, such as graphs and ROC curves, through a simple and interactive, yet statistically rigorous, workflow (12). CombiROC was initially developed to refine marker combinations from diverse omics data and has since been applied to other outcomes, such as the detection of lung overload (13), plasma biomarkers (14), and gene markers (15). CombiROC was recently applied in COVID-19 research to improve classification by optimizing biomarker combinations (16). However, to date, no study has used CombiROC to identify the best combinations of variables to predict clinical improvement in COVID-19. Moreover, a direct comparison of CombiROC with machine-learning methods such as LASSO has yet to be performed. Therefore, this study aimed to compare LASSO and CombiROC approaches for selecting predictive variables at hospital admission that detect clinical improvement after 7 days in patients with COVID-19.

## 2 Methods

### 2.1 Study design

This study is a secondary cross-sectional analysis of the placebo group in a previous randomized clinical trial (RCT) (17) involving patients with COVID-19. The analysis aimed to identify clinical, laboratory, and blood markers at hospital admission that predict clinical improvement after 7 days. Clinical improvement was defined as a 2-point or greater increase on the World Health Organization (WHO) clinical progression scale. Two methods were used for the analysis: LASSO (11) and CombiROC (12). The study adheres to the Declaration of Helsinki and was approved by the Brazilian National Commission for Research Ethics (CAAE: 30662420.0.1001.0008) and the individual Ethics Committees of all participating sites. This trial is registered with the Brazilian Registry of Clinical Trials (REBEC: RBR-88bs9x) and ClinicalTrials.gov (NCT04561219), registration date April 19, 2020. The study design and reporting follow the STROBE guidelines (18). The data were assessed retrospectively on May 17, 2023, and the authors did not have access to information that could identify individual participants after data collection.

### 2.2 Patients

The analysis included consecutive patients with COVID-19 pneumonia admitted to 19 hospitals in Brazil from April 20 to October 15, 2020. Inclusion criteria were as follows: adult patients (≥18 years) requiring supplemental oxygen (SpO_2_ < 93%), admitted with COVID-19 symptoms, chest computed tomography findings suggestive of viral pneumonia, or a positive reverse transcriptase-polymerase chain reaction test for SARS-CoV-2. Available clinical, laboratory, and blood marker data were collected. Exclusion criteria were a history of severe liver disease, chronic kidney disease with estimated glomerular filtration rate <30 ml/min/1.73 m^2^, severe heart failure (New York Heart Association classes 3 and 4), severe chronic obstructive pulmonary disease (GOLD classes 3 and 4), cancer within the last 5 years, known autoimmune disease, or clinical suspicion of tuberculosis or bacterial pneumonia.

### 2.3 Data sources/measurements

#### 2.3.1 Demographic, clinical, and laboratory data at hospital admission

Demographic data (age and sex), clinical data (temperature, respiratory rate, heart rate, SpO_2_), and laboratory markers (hematocrit, hemoglobin, leukocytes, neutrophils, lymphocytes, platelets, C-reactive protein, ferritin, lactate dehydrogenase, troponin, and D-dimer) at admission were collected.

#### 2.3.2 Blood biomarkers at hospital admission

Blood samples were taken at admission, labeled with each patient’s unique identifier, and analyzed in local laboratories. Blood biomarkers were analyzed using a 48-plex cytokine screening panel (Bio-Plex Pro Human Cytokine Screening Panel, 48-Plex). The following mediators were analyzed: basic fibroblast growth factor, eotaxin, granulocyte colony-stimulating factor, granulocyte-macrophage colony-stimulating factor, interferon (IFN)-γ, interleukin (IL)-1β, IL-1ra, IL-1α, IL-2Rα, IL-3, IL-12 (p40), IL-16, IL-2, IL-4, IL-5, IL-6, IL-7, IL-8, IL-9, growth-related oncogene alpha, hepatocyte growth factor (HGF), IFN-α2, leukemia inhibitory factor, monocyte chemotactic protein (MCP)-3, IL-10, IL-12 (p70), IL-13, IL-15, IL-17A, IP-10, MCP-1, monokine induced by IFN-γ, nerve growth factor-β, stem cell factor, stem cell growth factor-β, stromal cell-derived factor-1α, macrophage inflammatory protein-1α and-1β, platelet-derived growth factor (PDGF)-BB, RANTES (regulated upon activation, normal T cell expressed and secreted), tumor necrosis factor (TNF)-α, vascular endothelial growth factor, T cell–attracting chemokine (CTACK), macrophage migration inhibitory factor, TNF-related apoptosis-inducing ligand, IL-18, macrophage colony-stimulating factor, and TNF-β.

### 2.4 Outcome variable

The outcome variable was clinical improvement at 7 days, defined as an increase of at least two points on the WHO scale (17).

### 2.5 Statistical analysis

No formal sample size calculation was conducted given the exploratory nature of the study. All data meeting the inclusion criteria were analyzed. Descriptive statistics (mean ± standard deviation or median with interquartile range) were used for clinical, laboratory, and blood biomarker data, classified into groups with and without clinical improvement. The Shapiro–Wilk test assessed normality between groups. Parametric variables were analyzed using Student’s t test, and non-parametric variables were analyzed with the Mann–Whitney test.

Predictor selection for LASSO and CombiROC analysis was based on variables with less than 25% missing data and statistical significance in bivariate analysis. LASSO regression was used to shrink less informative predictors toward zero, enhancing model interpretability and handling multicollinearity (11). The LASSO method penalizes the beta estimation algorithm, pushing parameter estimates toward zero or exactly zero. This results in a simpler model that retains only the most important variables, making interpretation and analysis easier. This technique is particularly useful for reducing the number of explanatory variables in a model and addressing the issue of multicollinearity in data (19, 20). CombiROC (12) was used to evaluate various combinations of receiver operating characteristic (ROC) curves to identify the optimal biomarker combination for predicting clinical improvement. Furthermore, the combi function of the combiroc package calculates marker combinations and counts the positive samples for each class of the dependent variable based on a predefined threshold. A sample is considered positive for a given combination if its value exceeds the threshold for at least a specified number of markers within that combination. The threshold value is determined based on the recommendation of the markers distribution function, which corresponds to the threshold associated with the highest Youden Index (21). The code for the CombiROC and LASSO analysis, written in the R environment, is given in the Supplementary files.

Due to differing distributions among blood markers, data were normalized using the rescale function in the “scales” package (22). The optimal threshold for different biomarker combinations was selected using the Youden Index. ROC areas were compared with DeLong’s algorithm (23), using the roc.test function from the “pROC” package (24). Statistical significance was set at *p* < 0.05. All analyses were performed in the R 4.0.4 environment (22).

## 3 Results

**Figure 1:**
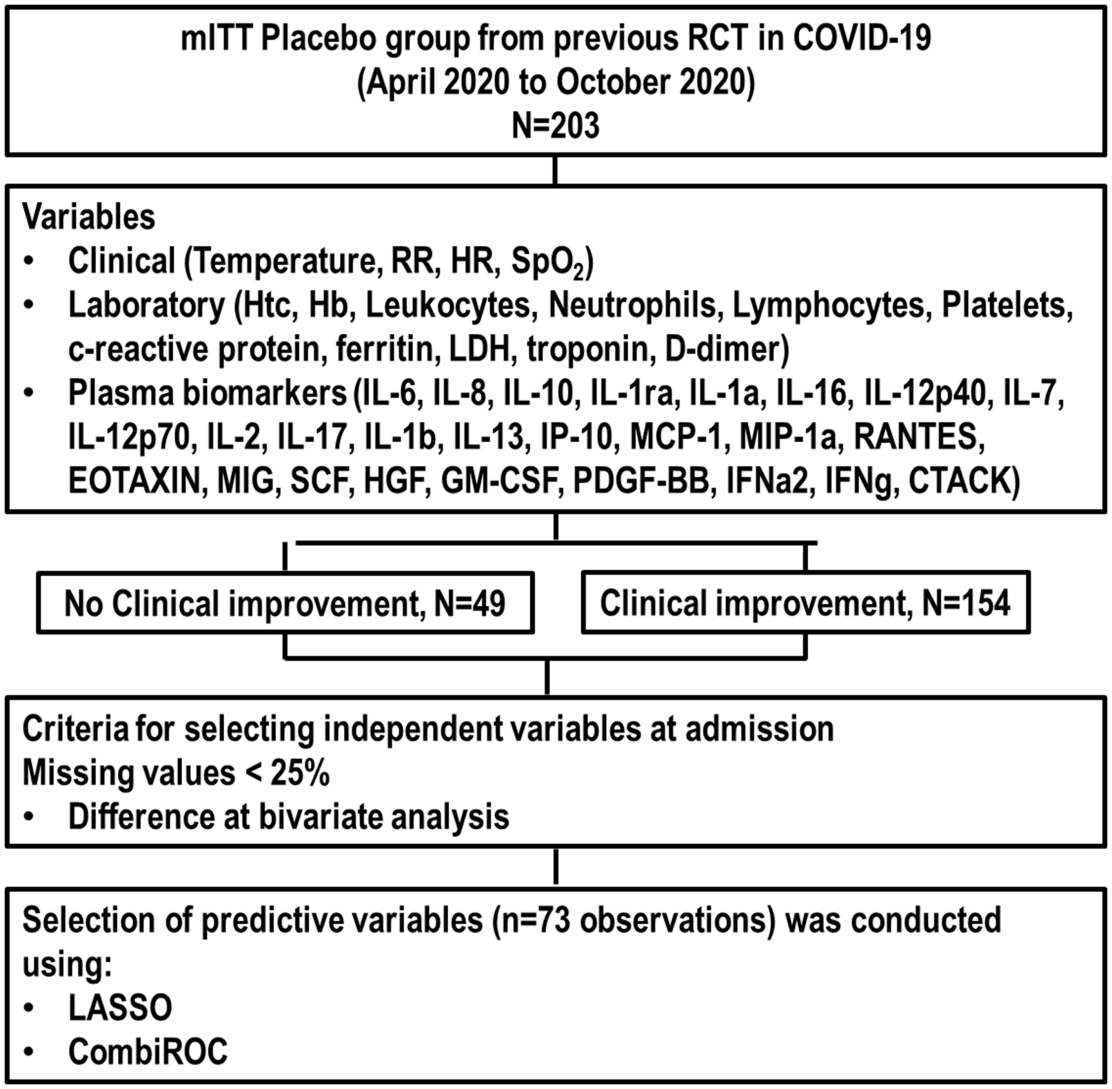
Flowchart of the study. CTACK, T cell–attracting chemokine; GM-CSF, granulocyte-macrophage colony-stimulating factor; Hb, hemoglobin; HGF, hepatocyte growth factor; HR, heart rate; Htc, hematocrit; IFN, interferon; IL, interleukin; LDH, lactate dehydrogenase; MCP, monocyte chemotactic protein; MIG, monokine induced by IFN-γ; MIP, macrophage inflammatory protein; mITT, modified intention-to-treat; PDGF, platelet-derived growth factor; RCT, randomized clinical trial; RR, respiratory rate; SCF, stem cell factor.

A total of 203 patients were included in the analysis (Figure 1), divided into two groups: clinical improvement (*n* = 154) and no clinical improvement (*n* = 49). The median age of patients in the intensive care unit was 55 years (interquartile range, 46–66 years); 65% were male. Patients in the clinical improvement group were generally older and had a lower respiratory rate (RR) but a higher level of oxygen saturation (SaO_2_), hematocrit, hemoglobin, platelets, IL-1ra, IL-13, RANTES, HGF, PDGF-BB, and CTACK compared with the no clinical improvement group (Table 1).

**Table 1:** Characteristics of the population at hospital admission.

Characteristics of the population at hospital admission	No.	All patients	No clinical improvement	Clinical improvement	*p* value between groups^*^
Absolute and relative frequencies, *n* (%)		203	49 (24)	154 (76)	
Age (years), median [IQR]	203	56 [46–66]	59 [52–66]	55 [45–65]	0.005
Sex, *n* (%)	203				
Male		131 (65)	30 (23)	101 (77)	0.701
Female		72 (35)	19 (26)	53 (74)	
Temperature (°C), median [IQR]	203	36.5 [36–37]	36.5 [36–37]	36.5 [36–37]	0.577
RR (bpm), median [IQR]	203	21 [19–24]	24 [20–29]	20 [19–23]	<0.001
HR (bpm), median [IQR]	203	87 [78–98]	90 (79–100)	86 (78–95)	0.581
SpO_2_ (%), median [IQR]	203	92 [91–93]	90 [86–92]	92 [92–93]	<0.001
Hematocrit (%), median [IQR]	203	40 [36–43]	37.8 [33.2–42.2]	40.9 [37.2–43.4]	0.004
Hemoglobin (mg/dl), median [IQR]	203	13.4 [12.1–14.6]	13.0 [10.8–14.2]	13.5 [12.4–14.7]	0.011
Leukocytes (cells/μl), median [IQR]	203	7,500 [5850–9,795]	7,840 [6100–10,900]	7,500 [5732–9,643]	0.254
Neutrophils (cells/μl), median [IQR]	201	5,538 [4086–7,250]	5,960 [4278–9,413]	5,442 [3988–7,026]	0.149
Lymphocytes (cells/μl), median [IQR]	203	1,920 [1367–2,610]	1,833 [1369–2,620]	1,937 [1368–2,587]	0.833
Platelets (10^3^/μl), median [IQR]	203	232 [165–289]	191 [152–256]	227 [169–300]	0.022
C-reactive protein (mg/L), median [IQR]	203	121 [85–149]	126 [96–146]	114 [79–151]	0.370
Ferritin (mg/L), median [IQR]	196	443 [260–746]	436 [294–700]	445 [257–793]	0.880
LDH (IU/L), median [IQR]	191	267 [176–394]	277 [171–395]	265 [180–391]	0.787
Troponin (mg/dl), median [IQR]	203	0.02 [0.01–0.05]	0.02 [0.01–0.05]	0.02 [0.01–0.05]	0.706
D-dimer (mg/dl), median [IQR]	191	1,010 [483–1727]	1,024 [490–1,429]	1,010 [486–2053]	0.678
IL-6	115	14.0 [9.5–65.3]	12.5 [11.0–30.5]	15.5 [8.5–67.6]	0.724
IL-8	106	19.6 [12.0–196.4]	13.0 [10.0–96.8]	21.4 [13.0–214.4]	0.123
IL-10	139	56.4 [14.0–635.5]	18.0 [13.0–299.6]	73.6 [14.0–718.9]	0.148
IL-1rα	107	977.6 [12.0–1580.3]	14.0 [10.8–1144.4]	977.6 [13.8–1580.3]	0.04
IL-1α	105	76.9 [13.5–811.2]	15.0 [12.0–257.9]	174.9 [14.0–815.7]	0.08
IL-16	105	138.9 [15.0–550.9]	16.5 [13.1–505.9]	329.2 [16.0–540.1]	0.079
IL-12p40	108	810.7 [11.0–2379.6]	14.0 [10.5–2397.8]	1286.8 [11.0–2379.6]	0.309
IL-7	107	219.6 [12.0–2175.4]	14.5 [10.5–668.9]	701.9 [12.0–2665.8]	0.059
IL-12p70	114	17.0 [13.3–78.1]	14.0 [12.5–47.1]	19.0 [14.0–78.1]	0.101
IL-2	102	32.4 [13.0–209.5]	13.5 [12.2–120.6]	41.3 [13.3–225.0]	0.111
IL-17	107	32.7 [14.0–299.8]	15.5 [13.0–106.2]	74.8 [15.0–305.3]	0.059
IL-1β	122	64.8 [14.0–839.2]	14.5 [13.0–331.1]	107.8 [14.6–978.8]	0.053
IL-13	106	106.8 [9.0–342.5]	9.0 [7.0–147.2]	160.7 [9.0–382.0]	0.013
IP-10	132	421.1 [43.0–2062.6]	285.2 [38.0–2275.5]	554.8 [45.5–1964.2]	0.733
MCP-1	114	70.7 [14.0–246.7]	17.3 [13.0–84.4]	79.0 [15.0–251.4]	0.055
MIP-1α	123	17.0 [8.4–27.9]	15.5 [12.0–26.1]	17.0 [7.9–27.9]	0.747
RANTES	108	152.7 [15.0–1103.3]	15.0 [13.8–801.2]	259.8 [18.8–1103.3]	0.02
EOTAXIN	129	20.6 [11.4–284.3]	13.0 [10.8–124.6]	47.4 [12.5–324.1]	0.065
MIG	106	204.1 [11.6–681.0]	13.0 [10.5–409.4]	250.1 [12.0–687.6]	0.073
SCF	104	25.8 [12.0–653.9]	14.0 [11.3–104.8]	54.2 [13.6–725.2]	0.11
HGF	118	498.9 [18.0–1851.1]	18.0 [14.4–1070.5]	578.7 [19.3–1992.2]	0.039
GM-CSF	116	12.0 [7.1–27.4]	10.0 [5.7–13.5]	13.3 [8,1–31.2]	0.149
PDGF-BB	115	639.2 [16.0–4454.1]	16.5 [14.0–2044.8]	837.9 [17.6–4611.5]	0.036
IFNα2	107	108.7 [11.5–304.9]	12.0 [9.8–224.8]	144.7 [12.8–304.9]	0.072
IFNg	113	22.2 [9.2–297.7]	11.5 [9.0–34.7]	53.3 [11.0–297.7]	0.061
CTACK	103	254.5 [13.3–1164.7]	14.5 [12.0–579.8]	337.8 [15.0–1324.2]	0.045

The descriptive analysis of the data is presented as absolute frequencies (n) and percentages according to the group. No. is the number of values gathered according to the respective variables. See the list of abbreviations at the end of the text for the definitions of the abbreviations used in the table. RR: respiratory rate, HR: heart rate, SpO_2_: peripheral oxygen saturation, LDH: lactate dehydrogenase, Interleukin (IL)-1β, IL-1α, IL-1rα, IL-2, IL-12 (p40), IL-6, IL-7, IL-8, IL-10, IL-12 (p70), IL-13, IL-16, IL-17, IP-10: Interferon gamma-induced protein 10, MCP-1: Monocyte Chemoattractant Protein-1, MIP-1α: Macrophage Inflammatory Protein-1 Alpha, RANTES: regulated upon activation, normal T cell expressed, and secreted, SCF: stem cell factor, HGF: hepatocyte growth factor, GM-CSF: Granulocyte-macrophage colony-stimulating factor, PDGF-BB: platelet-derived growth factor, IFN: interferon, CTACK: T cell–attracting chemokine.

* Mann–Whitney U test, Student’s *t* test or *χ*^2^ test (*p* < 0.05).

After applying the selection criteria (missing values <25% and significant differences in bivariate analysis), predictive variables were age, RR, SaO_2_, hematocrit, hemoglobin, platelets, IL-1ra, IL-13, RANTES, HGF, PDGF-BB, and CTACK across 73 observations. The individual predictive performance metrics (sensitivity, specificity, accuracy, and odds ratio [OR]) of these variables predicting clinical improvement are presented in Table 2.

**Table 2:** Individual performances of the LASSO selected variables and the top five best combination of variables according to the AUC to predict clinical improvement (≥2 points on the WHO scale).

Variables	AUC (95% CI)	Sensitivity, % (95% CI)	Specificity, % (95% CI)	Accuracy (%)	OR (95% CI)
IL-1ra	0.637 (0.482–0.791)	69 (54–80)	58 (33–80)	66	2.93 (1.00–9.02)
IL-3	0.635 (0.497–0.772)	26 (15–40)	100 (82–100)	45	Inf (1.37–inf)
RANTES	0.646 (0.501–0.790)	69 (54–80)	63 (38–84)	67	3.63 (1.22–11.55)
HGF	0.615 (0.462–0.769)	61 (47–74)	68 (43–87)	63	3.31 (1.11–10.92)
PDGF-BB	0.65 (0.502–0.797)	65 (51–77)	68 (43–87)	66	3.87 (1.29–12.84)
CTACK	0.606 (0.461–0.751)	32 (20–46)	90 (67–99)	47	3.64 (0.89–27.10)
RR	0.594 (0.433–0.755)	57 (43–71)	63 (38–84)	59	2.27 (0.78–7.08)
SaO_2_	0.715 (0.580–0.851)	74 (60–85)	63 (38–84)	71	4.74 (1.57–15.39)
Htc	0.67 (0.530–0.809)	76 (62–87)	53 (29–76)	70	3.42 (1.13–10.67)
Hg	0.602 (0.439–0.765)	87 (75–95)	37 (16–62)	74	3.82 (1.09–13.64)
Platelets	0.569 (0.428–0.711)	59 (45–72)	63 (38–84)	60	2.44 (0.84–7.64)
LASSO					
SaO_2_, Htc, IL-13	0.704 (0.571–0.837)	98 (92–100)	26 (10–48)	82	13.19 (2.67–106.54)
CombiROC					
CTACK, Hb, HGF, Htc, IL-3, PDGF-BB, RANTES, SaO_2_	0.823 (0.708–0.937)	82 (69–91)	74 (49–91)	80	11.56 (3.53–44.25)
Hb, HGF, Htc, IL-3, PDGF-BB, RANTES, SaO_2_	0.823 (0.709–0.936)	82 (69–91)	74 (49–91)	80	11.56 (3.53–44.25)
Hb, HGF, Htc, IL-1ra, IL-3, PDGF-BB, RANTES, SaO_2_	0.823 (0.709–0.936)	80 (66–89)	74 (49–91)	79	10.31 (3.19–38.95)
CTACK, RR, Hb, HGF, Htc, IL-1ra, IL-3, PDGF-BB, RANTES, SaO_2_	0.822 (0.708–0.936)	82 (69–91)	74 (49–91)	80	11.56 (3.53–44.25)
CTACK, RR, Hb, HGF, Htc, IL-1ra, IL-3, RANTES, SaO_2_	0.821 (0.706–0.935)	80 (66–89)	74 (49–91)	78	10.31 (3.19–38.95)

OR represents the odds that an outcome will occur given a particular exposure, compared with the odds of the outcome occurring in the absence of that exposure. If the outcome is the same in both groups, the ratio is 1, which implies there is no difference between the two arms of the study, however, if the OR is >1, the control is better than the intervention. If the OR is <1, the intervention is better than the control. The 95% confidence interval (CI) is used to estimate the precision of the OR. A large CI indicates a low level of precision of the OR, whereas a small CI indicates a higher precision of the OR. The OR was calculated considering the confusion matrix of each model, generated from the cut-off specified by the Youden Index. See the list of abbreviations at the end of the text for the definitions of the abbreviations used in the table.

**Figure 2:**
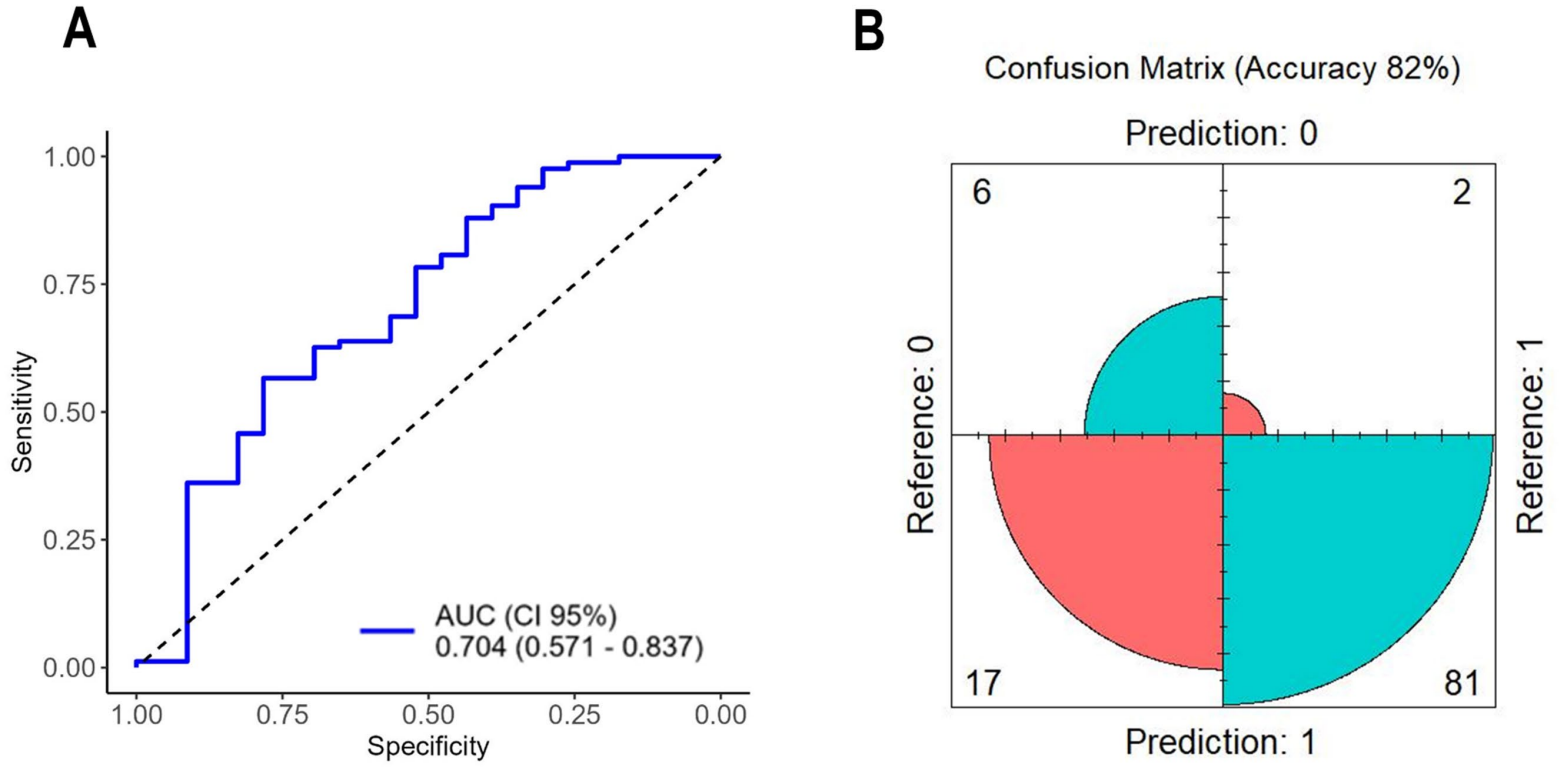
**(A)** ROC curve of variables selected by LASSO; **(B)** confusion matrix of variables selected by LASSO. AUC, area under the curve; CI, confidence interval.

LASSO analysis identified SaO_2_, hematocrit, and IL-13 as the three key predictive variables, achieving an area under the curve (AUC) of 0.704 (95% CI, 0.571–0.837) (Figure 2A). These variables demonstrated a sensitivity of 98%, specificity of 26%, accuracy of 82%, and OR of 13.19 (Table 2), with a confusion matrix indicating 17 false positives, contributing to low specificity (Figure 2B).

**Figure 3:**
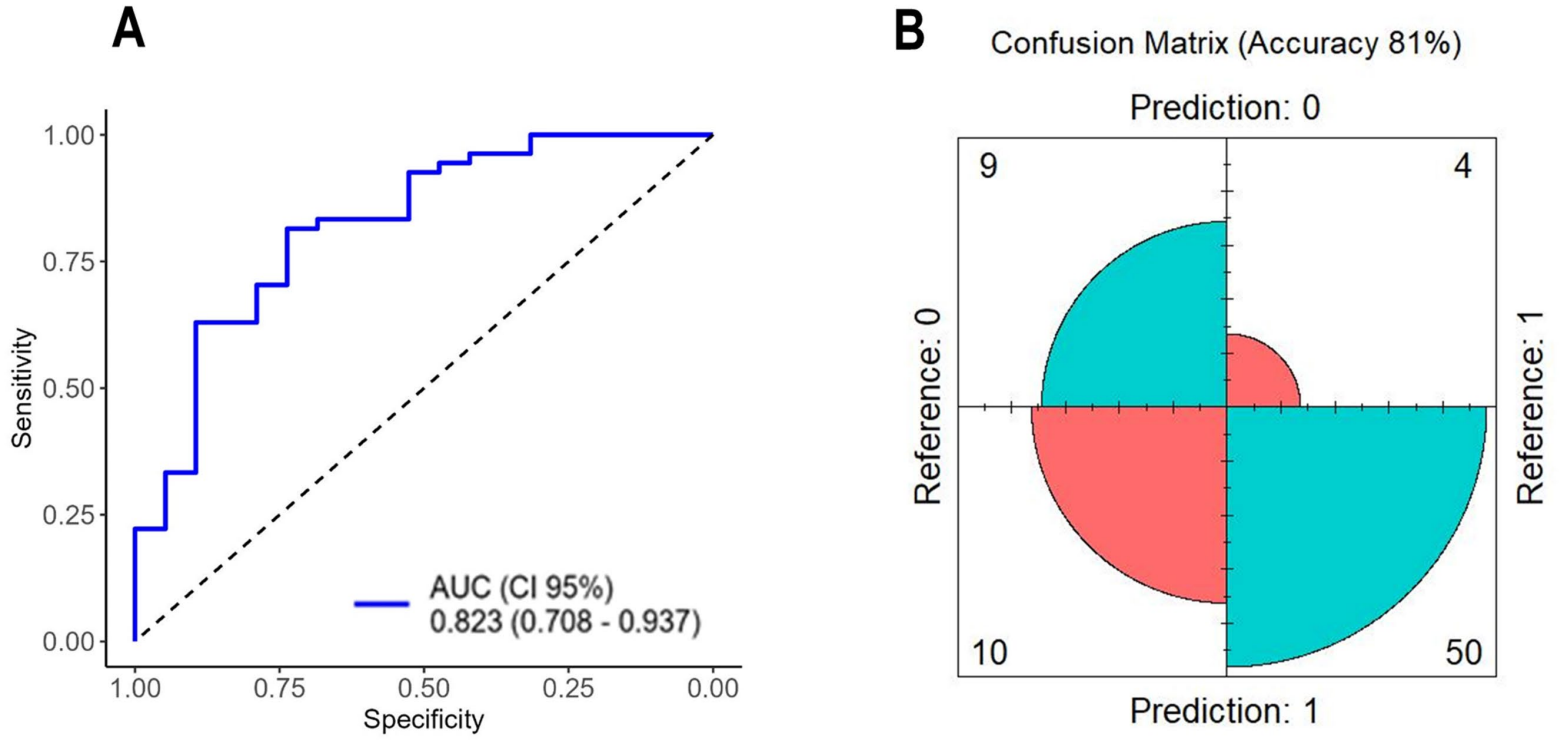
**(A)** ROC curve of variables selected by CombiROC; **(B)** confusion matrix of variables selected by CombiROC. AUC, area under the curve; CI, confidence interval.

A total of 2036 combinations were tested in the CombiROC analysis; the top five performing combinations are highlighted in Table 2. The best CombiROC combination (CTACK, Hb, HGF, Htc, IL-3, PDGF-BB, RANTES, and SaO_2_) achieved an AUC of 0.823 (95% CI, 0.708–0.937) (Figure 3A) with sensitivity of 82%, specificity of 74%, accuracy of 80%, and OR of 11.56. The confusion matrix showed 10 false positives, yielding moderate specificity (Figure 3B).

**Figure 4:**
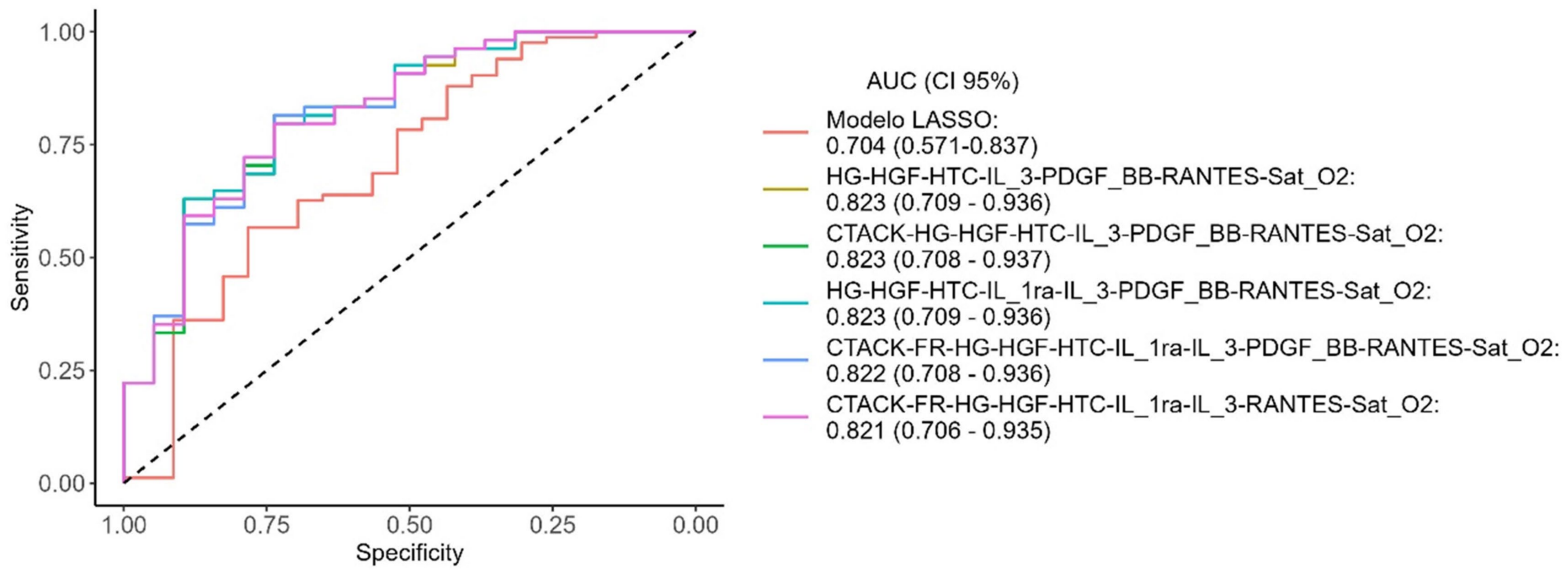
ROC curves of the LASSO model and the top 5 obtained after CombiROC analysis. AUC, area under the curve; CI, confidence interval.

ROC curves for the variables selected by LASSO and the top five CombiROC combinations are shown in Figure 4. No significant differences were observed between the LASSO model and the top five CombiROC models based on DeLong’s test (*p* = 0.185, *p* = 0.187, *p* = 0.186, *p* = 0.190, and *p* = 0.194, respectively).

## 4 Discussion

In patients hospitalized with COVID-19 pneumonia, we found that (1) the three predictive variables by LASSO (SaO_2_, hematocrit, and IL-13) demonstrated high sensitivity but low specificity in predicting clinical improvement; (2) the best combination of markers selected by CombiROC included more variables (CTACK, Hb, HGF, hematocrit, IL-3, PDGF-BB, RANTES, SaO_2_) and showed balanced sensitivity and specificity; (3) LASSO and CombiROC analyses yielded similar accuracy, with comparable ROC curves. This method is relevant for predicting clinical improvement or deterioration in non-COVID-19 acute respiratory distress syndrome (25). Nevertheless, there are important challenges to be overcome, such as data availability and the development and deployment of AI models.

We chose to use a modified intention-to-treat placebo group from a previous RCT (17) to better reflect the natural progression of the disease without treatment bias. This approach allowed us to assess the primary outcome at 7 days post hospital admission, capturing a period marked by significant clinical symptoms and lung inflammation due to SARS-CoV-2 infection (26). We used WHO clinical status for improvement as the primary outcome, a widely validated measure (27). Clinical improvement was defined as at least a 2-point increase in WHO clinical status. This outcome has been used in clinical trials dealing with pharmacological treatment for patients with COVID-19 (28). The WHO Clinical Progression Scale has been developed to facilitate data pooling across cohort studies and clinical trials, with the objective of expediting the exchange of knowledge to benefit patients infected with SARS-CoV-2 and to inform optimal resource planning (29). We also selected general clinical and laboratory data commonly used worldwide, ensuring the findings can be externally validated in future studies. Plasma biomarkers were chosen pragmatically from a multiplex kit of 47 cytokine markers, most of which are relevant to early COVID-19 pathophysiology. However, we excluded biomarkers with less than 50% data availability, because this would compromise the predictive analysis.

CombiROC, initially developed as a web-based tool for selecting optimal omics markers (12), has been applied for predicting lung overload in COVID-19 (13). CombiROC enables interactive selection of optimal marker combinations and generates visual feedback such as ROC curves. In our analysis, CombiROC required eight variables (CTACK, Hb, HGF, hematocrit, IL-13, PDGF-BB, RANTES, and SaO_2_) to achieve a balanced discriminative analysis, yielding 82% accuracy with moderate sensitivity (82%) and specificity (74%). However, these markers are more complex to assess at admission, potentially limiting feasibility in routine hospital settings. Notable biomarkers included in the CombiROC analysis, such as CTACK, HGF, and PDGF-BB, reflect distinct aspects of the immune response. CTACK is associated with T cell homing to lung tissues and may play a role in early inflammatory responses (30). HGF, produced by mesenchymal cells, functions as a regulator of the immune response and tissue repair; it may indicate early lung recovery in patients with COVID-19 (31, 32). IL-13, a central mediator of airway responsiveness, may reduce ACE2 expression on epithelial cells, potentially influencing viral replication dynamics (33, 34). PDGF-BB and RANTES (CCL5) have been associated with milder disease and may serve as markers of early immune responses conducive to recovery (35–37).

From a statistical point of view, both methods, CombiROC and LASSO, showed equivalent performance in predicting clinical improvement. Nevertheless, from a clinical point of view, there are some important insights about the discriminatory capacity of both methods. As observed, although CombiROC selected more variables, it showed good balance in recognizing those patients with COVID-19 who will or will not clinically improve. It can be inferred that if we are dealing with a population of patients with COVID-19 with very little clinical and laboratory information, it might be interesting to use the variables selected by the CombiROC analysis. This could maximize the prediction of clinical improvement or no clinical improvement because both truly positive cases and truly negative cases will be recognized. This can help the decision-making and ultimately improve healthcare for patients (38). On the other hand, if we are dealing with a population of patients with COVID-19 with some clinical information, such as age (39), absence of co-morbidity (40), presence of vaccine (41), we may use fewer and simpler variables selected by the LASSO analysis to identify likely truly positive case for clinical improvement; LASSO variables will detect with good sensitivity (98%). In practice, the process of selecting a discrete threshold value for a given test must carefully weigh the relative importance of a high true positive rate versus a high true negative rate and, by extension, the consequences of false negative and false positive results for the particular test (38).

### 4.1 Limitations

Our study has several limitations. First, the number of patients with a poor outcome was low, limiting the analysis. Second, as a secondary analysis, the SARS-CoV-2 strain was from the early wave of the pandemic, before widespread vaccination efforts. By 2023, >13 billion vaccine doses had been administered globally, although coverage remains low in some regions, particularly low-income areas where it is estimated to be under 30% (42). We did not assess plasma biomarkers on day 7 due to limited sample size and kit limitations. There are additional classification models, such as gradient boosting, hist gradient boosting, multilayer perceptron, among others that could be used to predict clinical improvement in COVID-19 patients (43).

## 5 Conclusion

In patients hospitalized with COVID-19 pneumonia, LASSO and CombiROC analyses showed comparable accuracy and ROC curve performance in predicting clinical improvement. LASSO identified three primary variables (SaO_2_, hematocrit, and IL-13) that yielded high sensitivity but low specificity, whereas CombiROC, with eight variables (CTACK, Hb, HGF, hematocrit, IL-3, PDGF-BB, RANTES, SaO2), provided a balanced sensitivity and specificity for predicting improvement. Thus, in patients with COVID-19, SaO_2_, hematocrit, and IL-13 may predict clinical improvement.

## Data Availability

The raw data supporting the conclusions of this article will be made available by the authors, without undue reservation.

## Glossary

AI: Artificial intelligence
AUC: Area under the curve
CI: Confidence interval
CTACK: T cell–attracting chemokine
Hb: Hemoglobin
Hct: Hematocrit
HGF: Hepatocyte growth factor
IFN: Interferon
IL: Interleukin
IQR: Interquartile range
LASSO: Least absolute shrinkage and selection operator
LDH: Lactate dehydrogenase
MCP: Monocyte chemotactic protein
MIG: Monokine induced by IFN-γ
OR: Odds ratio
PDGF: Platelet-derived growth factor
RCT: Randomized clinical trial
ROC: Receiver operating characteristic
RR: Respiratory rate
SCF: Stem cell factor
TNF: Tumor necrosis factor
WHO: World Health Organization
