# Supplementary material for: Predicting clinical outcomes at hospital admission of patients with COVID-19 pneumonia using artificial intelligence: a secondary analysis of a randomized clinical trial
Source: Front Med (Lausanne). 2025 May 2;12:1561980. doi: 10.3389/fmed.2025.1561980 (PMC12081340; doi:10.3389/fmed.2025.1561980)
Supplement: Supplementary file 2 [file Data_Sheet_1.docx]

Supplementary Material


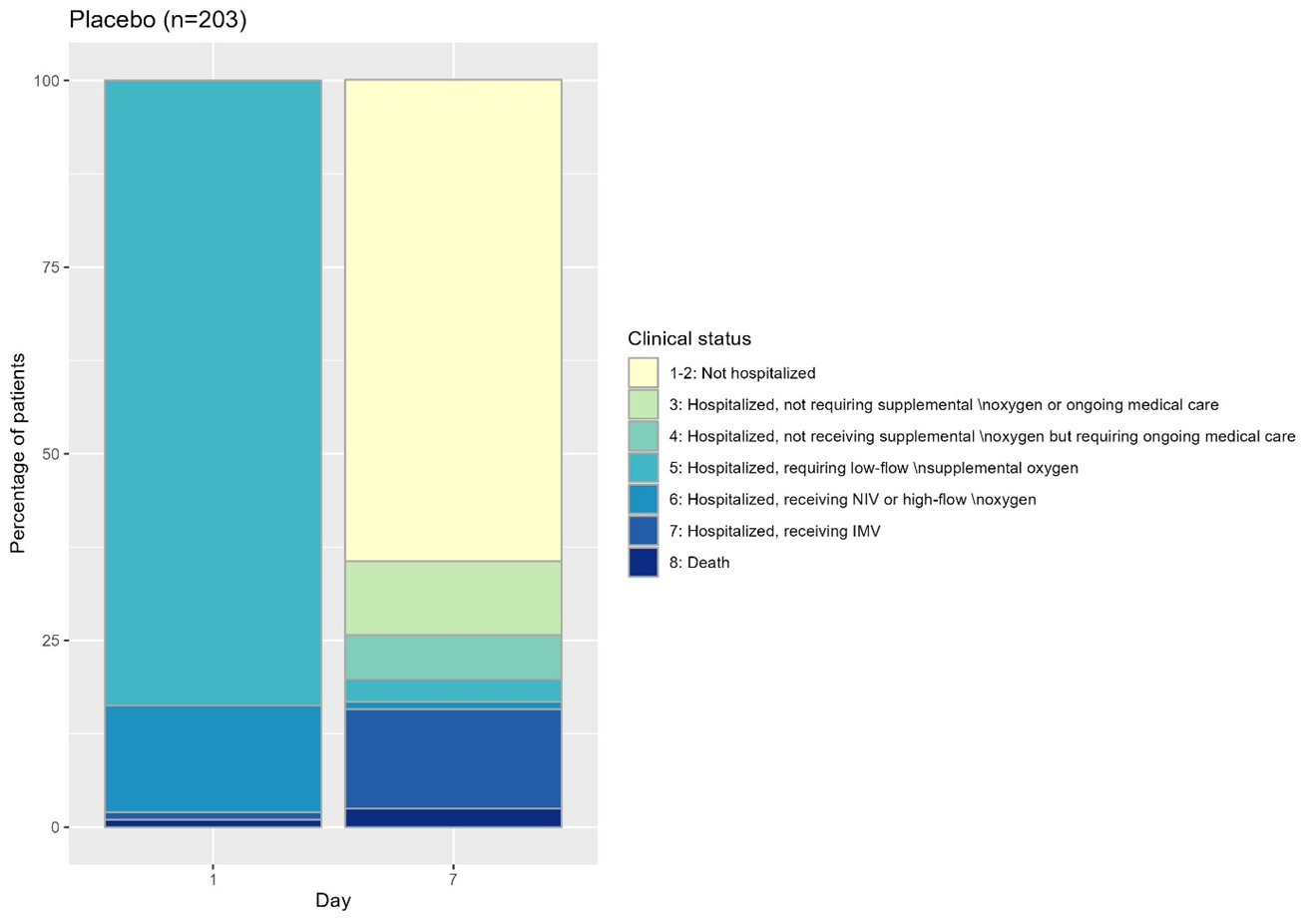


**Supplementary Figure 1**. WHO clinical scale of placebo group at day 1 and day 7.

**Supplemental Figure 2.**


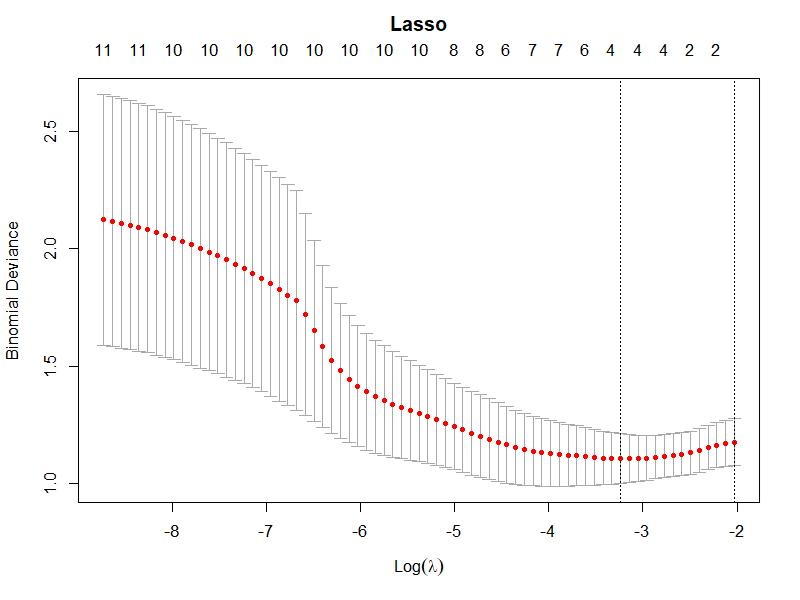


**Supplementary Figure 2**. Binomial deviance of the LASSO model with different log(λ).

# Supplementary File 3

#Pacotes

library(readxl)

library(tidyverse)

library(writexl)

library(reshape)

library(glmnet)

library(sjPlot)

library(pROC)

library(combiroc)

library(scales)

library(epiR)

# Dados

dados = read_excel("Dados.xlsx")

# Ajuste dos dados

dados$`B-FGF(D1)`<-as.numeric(gsub("[^0-9.-]", ".", dados$`B-FGF(D1)`))

dados$`CTACK(D1)`<-as.numeric(gsub("[^0-9.-]", ".", dados$`CTACK(D1)`))

dados$`EOTAXIN(D1)`<-as.numeric(gsub("[^0-9.-]", ".", dados$`EOTAXIN(D1)`))

dados$`G-CSF(D1)`<-as.numeric(gsub("[^0-9.-]", ".", dados$`G-CSF(D1)`))

dados$`GM-CSF(D1)`<-as.numeric(gsub("[^0-9.-]", ".", dados$`GM-CSF(D1)`))

dados$`GROa(D1)`<-as.numeric(gsub("[^0-9.-]", ".", dados$`GROa(D1)`))

dados$`HGF(D1)`<-as.numeric(gsub("[^0-9.-]", ".", dados$`HGF(D1)`))

dados$`IFNg(D1)`<-as.numeric(gsub("[^0-9.-]", ".",dados$`IFNg(D1)`))

dados$`IL-10(D1)`<-as.numeric(gsub("[^0-9.-]", ".",dados$`IL-10(D1)`))

dados$`IL-12p40(D1)`<-as.numeric(gsub("[^0-9.-]", ".",dados$`IL-12p40(D1)`))

dados$`IL-12p70(D1)`<-as.numeric(gsub("[^0-9.-]", ".",dados$`IL-12p70(D1)`))

dados$`IL-13(D1)`<-as.numeric(gsub("[^0-9.-]", ".",dados$`IL-13(D1)`))

dados$`IL-17(D1)`<-as.numeric(gsub("[^0-9.-]", ".",dados$`IL-17(D1)`))

dados$`IL-1a(D1)`<-as.numeric(gsub("[^0-9.-]", ".",dados$`IL-1a(D1)`))

dados$`IL-2(D1)`<-as.numeric(gsub("[^0-9.-]", ".",dados$`IL-2(D1)`))

dados$`IL-2Ra(D1)`<-as.numeric(gsub("[^0-9.-]", ".",dados$`IL-2Ra(D1)`))

dados$`IL-3(D1)` <-as.numeric(gsub("[^0-9.-]", ".",dados$`IL-3(D1)`))

dados$`IL-6(D1)` <-as.numeric(gsub("[^0-9.-]", ".",dados$`IL-6(D1)`))

dados$`IL-9(D1)` <-as.numeric(gsub("[^0-9.-]", ".",dados$`IL-9(D1)`))

dados$`IP-10(D1)` <-as.numeric(gsub("[^0-9.-]", ".",dados$`IP-10(D1)`))

dados$`M-CSF(D1)` <-as.numeric(gsub("[^0-9.-]", ".",dados$`M-CSF(D1)`))

dados$`MCP-3(D1)` <-as.numeric(gsub("[^0-9.-]", ".",dados$`MCP-3(D1)`))

dados$`SCF(D1)` <-as.numeric(gsub("[^0-9.-]", ".",dados$`SCF(D1)`))

dados$`SDF-1a(D1)` <-as.numeric(gsub("[^0-9.-]", ".",dados$`SDF-1a(D1)`))

dados$`TNFa(D1)` <-as.numeric(gsub("[^0-9.-]", ".",dados$`TNFa(D1)`))

#########################################

#LASSO - SELECIONANDO APENAS AS VARIÁVEIS SIGNIFICATIVAS NA ANÁLISE DESCRITIVA

dados <- dados %>% mutate(melhora_clinica2 = ifelse(melhora_clinica == "Sim", 1, 0))

dados$melhora_clinica2= factor(dados$melhora_clinica2)

quali = dados %>% select(`IL-1ra(D1)`,`IL-13(D1)`,`RANTES(D1)`,`HGF(D1)`,

`PDGF-BB(D1)`, `CTACK(D1)`,

fr_d1, sat_o2, `htc D1`, hg_d1,

plaquet, melhora_clinica2)

quali= na.omit(quali)

#Regressão logística inicial

mod = glm(melhora_clinica2 ~ ., data = quali,family = binomial,maxit = 100000)

summary(mod)

## Regularização LASSO

#Formato das variáveis: dependente como vetor e independentes como matriz

x = model.matrix(melhora_clinica2 ~. , data = quali)

y = quali$melhora_clinica2

set.seed(100)

#Modelo LASSO

mod_lasso = cv.glmnet(x = x, y = y, family = "binomial", alpha = 1)

plot(mod_lasso); title("Lasso", line = 2.5)

mod_lasso$lambda.min

mod_lasso$lambda.1se

#Gráfico do LASSO

plot(mod_lasso$glmnet.fit, "lambda", label=FALSE)

#Modelo de regressão logística com as variáveis selecionadas pelo LASSO

quali = dados %>% select(sat_o2, `htc D1`, `IL-13(D1)`, melhora_clinica)

quali <- quali %>% mutate(melhora_clinica = ifelse(melhora_clinica == "Sim", 1, 0))

quali$melhora_clinica= factor(quali$melhora_clinica)

quali= na.omit(quali)

mod = glm(melhora_clinica ~ ., data = quali,family = binomial,maxit = 100000)

summary(mod)

tab_model(mod)

#Predição

quali$pred <- as.factor(

ifelse(

predict(mod,

newdata = quali,

type = "response")

>0.5,"1","0"))

#Matriz de confusão

mc= caret::confusionMatrix(quali$pred, quali$melhora_clinica, positive="1")

mc

fourfoldplot(mc$table, color = c("indianred1", "cyan3"),

conf.level = 0, margin = 2, main = "Confusion Matrix (Accuracy 82%)")

data <- as.table(matrix(c(81,17,2,6), nrow = 2, byrow = TRUE))

rval <- epi.tests(data, conf.level = 0.95)

print(rval)

epitools::oddsratio(data)

#Curva ROC do modelo

quali$pred2= predict(mod, newdata = quali, type = "response")

roc = roc(quali$melhora_clinica, quali$pred2,

smoothed = T, ci=T, ci.alpha=0.95, stratified=F, plot=T,

auc.polygon=T, max.auc.polygon=T, grid=T, print.auc= T, print.auc.y= 0.2, print.thres=T)

roclist = list('AUC (CI 95%) \n0.704 (0.571 - 0.837)' = roc)

ggroc(roclist, aes = "colour", size= 1) +

geom_segment(aes(x = 0, xend = 1, y = 1, yend = 0), color="black", linetype="dashed") +

labs(x = "Specificity", y = "Sensitivity", colour = "")+

theme_classic()+

scale_color_manual(values = c("blue", "red"))+

theme(axis.title.x = element_text(size = 12),

axis.text.x = element_text(size = 12),

axis.title.y = element_text(size = 12),

axis.text.y = element_text(size = 12),

legend.title = element_text(size = 12),

legend.text = element_text(size = 12),

legend.key.size= unit(1, 'cm'))

#############################################################################

#COMBIROc

df = dados %>% select(ID, melhora_clinica, `IL-1ra(D1)`,`IL-13(D1)`,

`RANTES(D1)`,`HGF(D1)`, `PDGF-BB(D1)`, `CTACK(D1)`,

fr_d1, sat_o2, `htc D1`, hg_d1, plaquet)

df$melhora_clinica[df$melhora_clinica == "Sim"] = "A"

df$melhora_clinica[df$melhora_clinica == "Não"] = "B"

colnames(df) = c("Patient.ID", "Class", "IL_1ra", "IL_3", "RANTES", "HGF", "PDGF_BB", "CTACK", "FR", "Sat_O2", "HTC", "HG", "Plaquetas")

df = na.omit(df)

#Normalizando as variáveis

df$IL_1ra= rescale(df$IL_1ra)

df$IL_3= rescale(df$IL_3)

df$RANTES= rescale(df$RANTES)

df$HGF= rescale(df$HGF)

df$PDGF_BB= rescale(df$PDGF_BB)

df$CTACK= rescale(df$CTACK)

df$FR= rescale(df$FR)

df$Sat_O2= rescale(df$Sat_O2)

df$HTC= rescale(df$HTC)

df$HG= rescale(df$HG)

df$Plaquetas= rescale(df$Plaquetas)

summary(df)

#Visão geral da distribuição dos marcadores

df_long = combiroc_long(df)

distr = markers_distribution(df_long, case_class = 'A',

y_lim = 0 , x_lim = 1,

signalthr_prediction = TRUE,

min_SE = 0, min_SP = 0,

boxplot_lim = 1)

distr$Boxplot

distr$ROC

coord = data.frame(distr$Coord) #Youden = SE + SE - 1

distr$Density_plot #Treshold = 0.24

distr$Density_summary

#Análise combinatória

combs <- combi(df, signalthr =0.24, combithr =1)

combs$names= dimnames(combs)[[1]]

#write_xlsx(combs, "Combinações e classificação_combiroc.xlsx")

#Resultados

reports <- roc_reports(data= df, markers_table= combs,

selected_combinations= c(1:2036),

single_markers=c("IL_1ra", "IL_3", "RANTES", "HGF", "PDGF_BB", "CTACK", "FR", "Sat_O2", "HTC", "HG", "Plaquetas"),

case_class='A')

metrics = data.frame(reports$Metrics)

metrics$names= dimnames(metrics)[[1]]

#Seleção das combinações com maior AUC

dimnames(combs)[[1]] = combs$Markers

reports2 <- roc_reports(data= df, markers_table= combs,

selected_combinations= c(1781, 1901, 1963, 2027, 1975),

single_markers=c("IL_1ra", "IL_3", "RANTES", "HGF", "PDGF_BB", "CTACK", "FR", "Sat_O2", "HTC", "HG", "Plaquetas"),

case_class='A')

reports2$Plot

############

#Comparação da curva roc do LASSO com os 5 melhores combinações

#

reports3 <- roc_reports(data= df, markers_table= combs,

selected_combinations= c(1781),

case_class='A')

comb1= data.frame(pred=reports3$Models$`HG-HGF-HTC-IL_3-PDGF_BB-RANTES-Sat_O2`$fitted.values,

melhora_clinica= df$Class)

comb1$melhora_clinica[comb1$melhora_clinica == "A"] = 1

comb1$melhora_clinica[comb1$melhora_clinica == "B"] = 0

comb1$melhora_clinica= factor(comb1$melhora_clinica)

roc1 = roc(comb1$melhora_clinica, comb1$pred,

smoothed = T, ci=T, ci.alpha=0.95, stratified=F, plot=T,

auc.polygon=T, max.auc.polygon=T, grid=T, print.auc= T, print.auc.y= 0.2, print.thres=T)

roc.test(roc, roc1, method = "delong")

#

reports3 <- roc_reports(data= df, markers_table= combs,

selected_combinations= c(1901),

case_class='A')

comb2= data.frame(pred=reports3$Models$`CTACK-HG-HGF-HTC-IL_3-PDGF_BB-RANTES-Sat_O2`$fitted.values,

melhora_clinica= df$Class)

comb2$melhora_clinica[comb2$melhora_clinica == "A"] = 1

comb2$melhora_clinica[comb2$melhora_clinica == "B"] = 0

comb2$melhora_clinica= factor(comb2$melhora_clinica)

roc2 = roc(comb2$melhora_clinica, comb2$pred,

smoothed = T, ci=T, ci.alpha=0.95, stratified=F, plot=T,

auc.polygon=T, max.auc.polygon=T, grid=T, print.auc= T, print.auc.y= 0.2, print.thres=T)

roc.test(roc, roc2, method = "delong")

#

reports3 <- roc_reports(data= df, markers_table= combs,

selected_combinations= c(1963),

case_class='A')

comb3= data.frame(pred=reports3$Models$`HG-HGF-HTC-IL_1ra-IL_3-PDGF_BB-RANTES-Sat_O2`$fitted.values,

melhora_clinica= df$Class)

comb3$melhora_clinica[comb3$melhora_clinica == "A"] = 1

comb3$melhora_clinica[comb3$melhora_clinica == "B"] = 0

comb3$melhora_clinica= factor(comb3$melhora_clinica)

roc3 = roc(comb3$melhora_clinica, comb3$pred,

smoothed = T, ci=T, ci.alpha=0.95, stratified=F, plot=T,

auc.polygon=T, max.auc.polygon=T, grid=T, print.auc= T, print.auc.y= 0.2, print.thres=T)

roc.test(roc, roc3, method = "delong")

#

reports3 <- roc_reports(data= df, markers_table= combs,

selected_combinations= c(2027),

case_class='A')

comb4= data.frame(pred=reports3$Models$`CTACK-FR-HG-HGF-HTC-IL_1ra-IL_3-PDGF_BB-RANTES-Sat_O2`$fitted.values,

melhora_clinica= df$Class)

comb4$melhora_clinica[comb4$melhora_clinica == "A"] = 1

comb4$melhora_clinica[comb4$melhora_clinica == "B"] = 0

comb4$melhora_clinica= factor(comb4$melhora_clinica)

roc4 = roc(comb4$melhora_clinica, comb4$pred,

smoothed = T, ci=T, ci.alpha=0.95, stratified=F, plot=T,

auc.polygon=T, max.auc.polygon=T, grid=T, print.auc= T, print.auc.y= 0.2, print.thres=T)

roc.test(roc, roc4, method = "delong")

#

reports3 <- roc_reports(data= df, markers_table= combs,

selected_combinations= c(1975),

case_class='A')

comb5= data.frame(pred=reports3$Models$`CTACK-FR-HG-HGF-HTC-IL_1ra-IL_3-RANTES-Sat_O2`$fitted.values,

melhora_clinica= df$Class)

comb5$melhora_clinica[comb5$melhora_clinica == "A"] = 1

comb5$melhora_clinica[comb5$melhora_clinica == "B"] = 0

comb5$melhora_clinica= factor(comb5$melhora_clinica)

roc5 = roc(comb5$melhora_clinica, comb5$pred,

smoothed = T, ci=T, ci.alpha=0.95, stratified=F, plot=T,

auc.polygon=T, max.auc.polygon=T, grid=T, print.auc= T, print.auc.y= 0.2, print.thres=T)

roc.test(roc, roc5, method = "delong")

#CURVA ROC DO LASSO E 5 MELHORES JUNTAS

roclist = list('Modelo LASSO: \n0.704 (0.571-0.837)' = roc,

'HG-HGF-HTC-IL_3-PDGF_BB-RANTES-Sat_O2: \n0.823 (0.709 - 0.936)' = roc1,

'CTACK-HG-HGF-HTC-IL_3-PDGF_BB-RANTES-Sat_O2: \n0.823 (0.708 - 0.937)' = roc2,

'HG-HGF-HTC-IL_1ra-IL_3-PDGF_BB-RANTES-Sat_O2: \n0.823 (0.709 - 0.936)' = roc3,

'CTACK-FR-HG-HGF-HTC-IL_1ra-IL_3-PDGF_BB-RANTES-Sat_O2: \n0.822 (0.708 - 0.936)' = roc4,

'CTACK-FR-HG-HGF-HTC-IL_1ra-IL_3-RANTES-Sat_O2: \n0.821 (0.706 - 0.935)' = roc5)

ggroc(roclist, aes = "colour", size= 0.7) +

geom_segment(aes(x = 0, xend = 1, y = 1, yend = 0), color="black", linetype="dashed") +

labs(x = "Specificity", y = "Sensitivity", colour = "Variáveis: AUC (CI 95%)")+

theme_classic()+

theme(axis.title.x = element_text(size = 12),

axis.text.x = element_text(size = 12),

axis.title.y = element_text(size = 12),

axis.text.y = element_text(size = 12),

legend.title = element_text(size = 12),

legend.text = element_text(size = 12),

legend.key.size= unit(1, 'cm'))

ggsave("ROC curve LASSO + combiroc.jpg", last_plot(), height = 4, width = 11, bg= "white")
